# Supplementary material for: Transcriptional Activation of a Pro-Inflammatory Response (NF-κB, AP-1, IL-1β) by the Vibrio cholerae Cytotoxin (VCC) Monomer through the MAPK Signaling Pathway in the THP-1 Human Macrophage Cell Line
Source: Int J Mol Sci. 2023 Apr 14;24(8):7272. doi: 10.3390/ijms24087272 (PMC10139130; doi:10.3390/ijms24087272)
Supplement: Supplementary file 1 [file ijms-24-07272-s001.zip › ijms-2278244-supplementary.pdf]

Supplementary Information 1

(A)

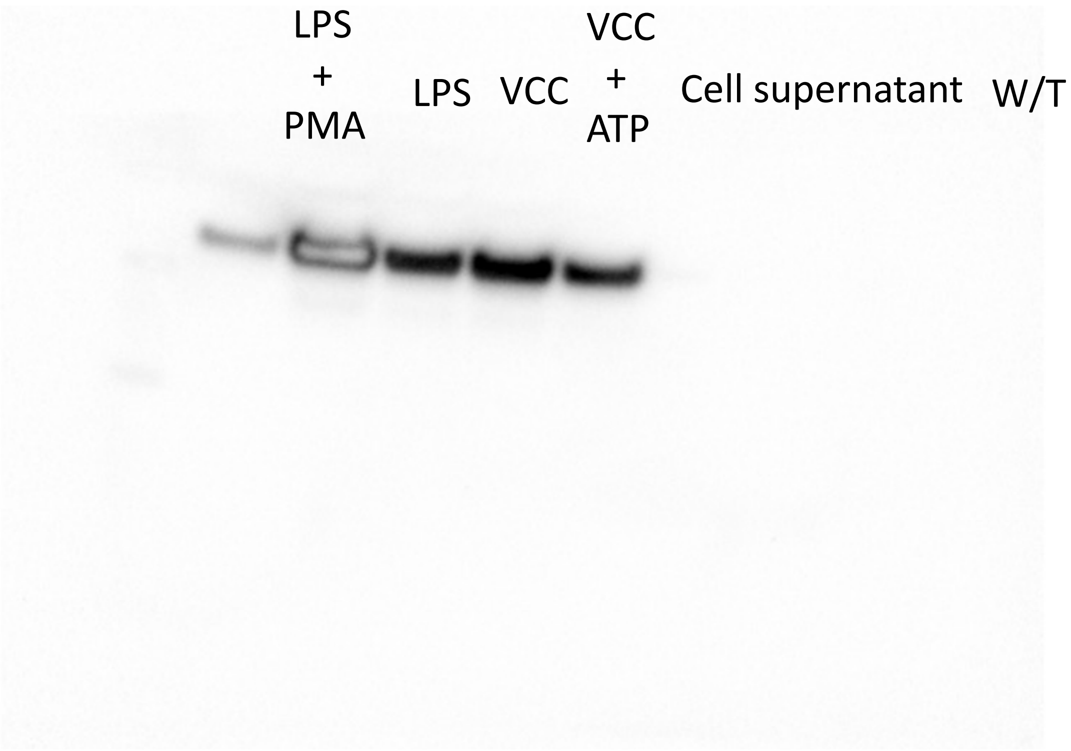

(B)

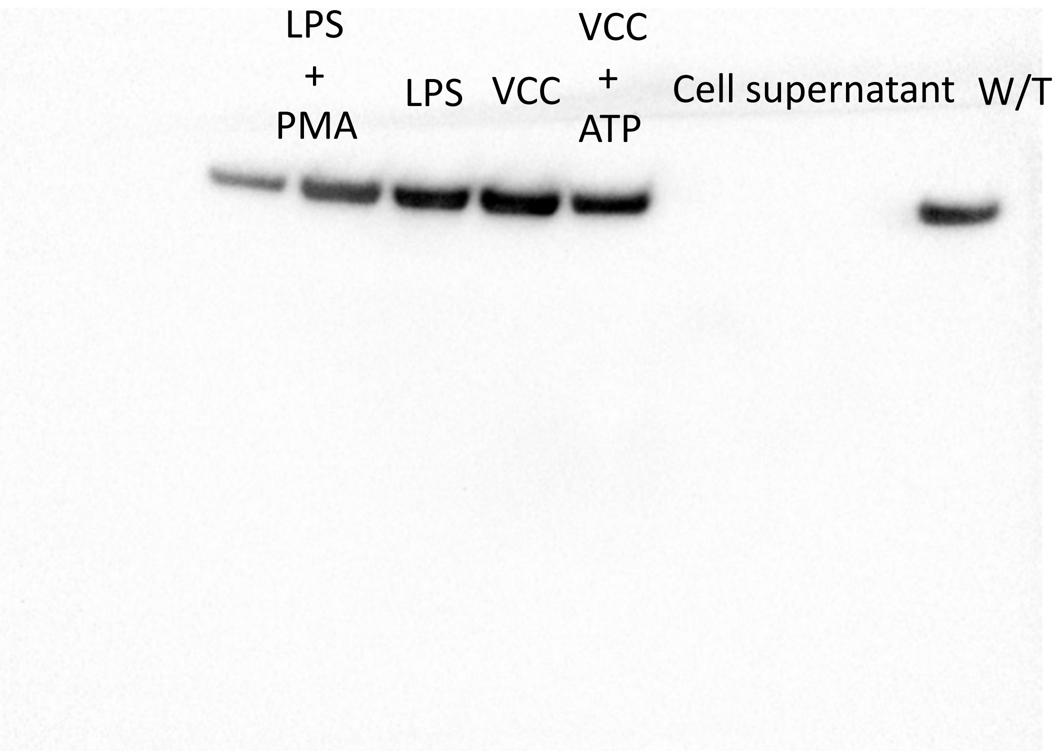

(A) Original Western blot showing the production of Pro IL- 1β by THP-1 macrophagers induced by treatments with 40 pg/ml of VCC after 6 h incubation, with no vacuolating effect. As well, expression of pro IL-1β induced by treatments with several inductors (LPS + PMA, LPS, VCC, VCC + ATP) 6 hours showing this in vitro system is appropriate to demonstrate activation of the IL- 1β by activation of the inflammasome. The negative control, a mock without treatment is named (W/T). (B) Original Western Blot of the β- actin protein, considered an internal control. Lipopolysaccharide (LPS), phorbol 12-myristate 13-acetate (PMA), *Vibrio cholerae* cytotoxin (VCC) and Adenosine

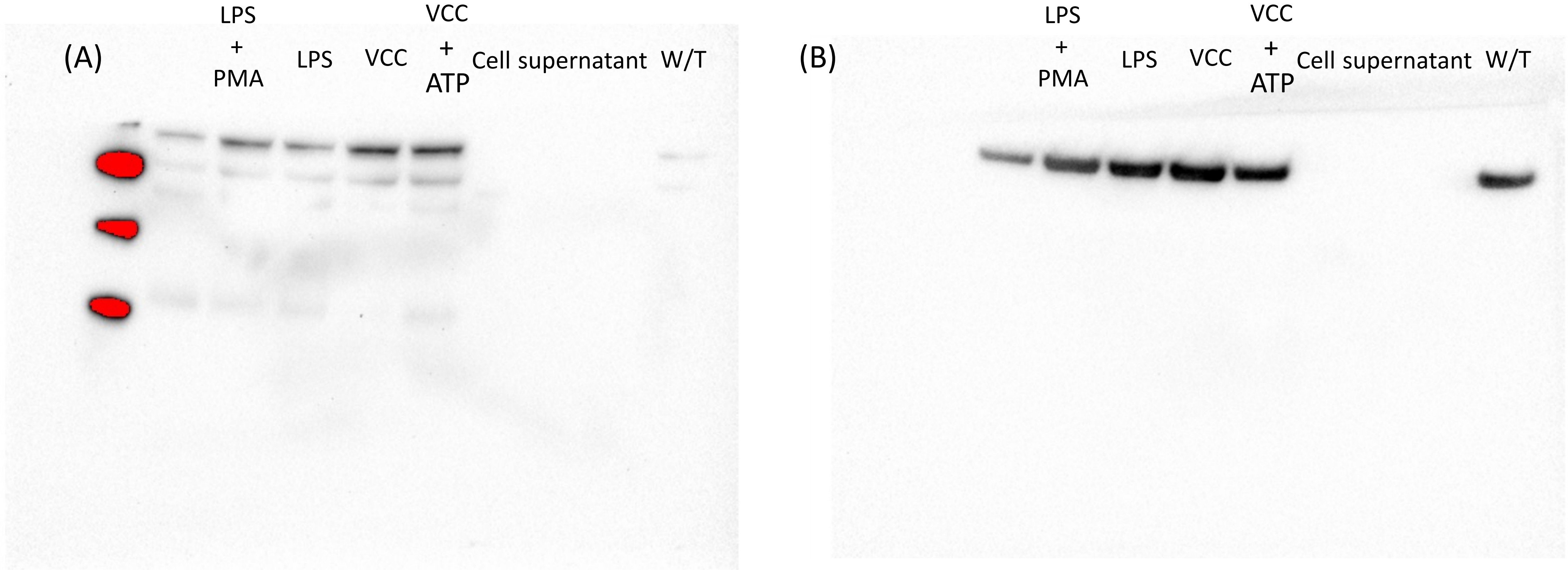

(A) Original Western blot showing mature Caspase-1 proteolysis processed, from treatments of THP-1 macrophages with 40 pg/ml of VCC (6 h incubation) showing no vacuolating effect. As well as proteolytic activated Caspase-1 from treatments with different inflammasome activators (LPS + PMA, LPS, VCC, VCC + ATP). Negative control, a mock without treatment was named (W/T). (B) Original Western Blot of β- actin, considered an internal control of protein.

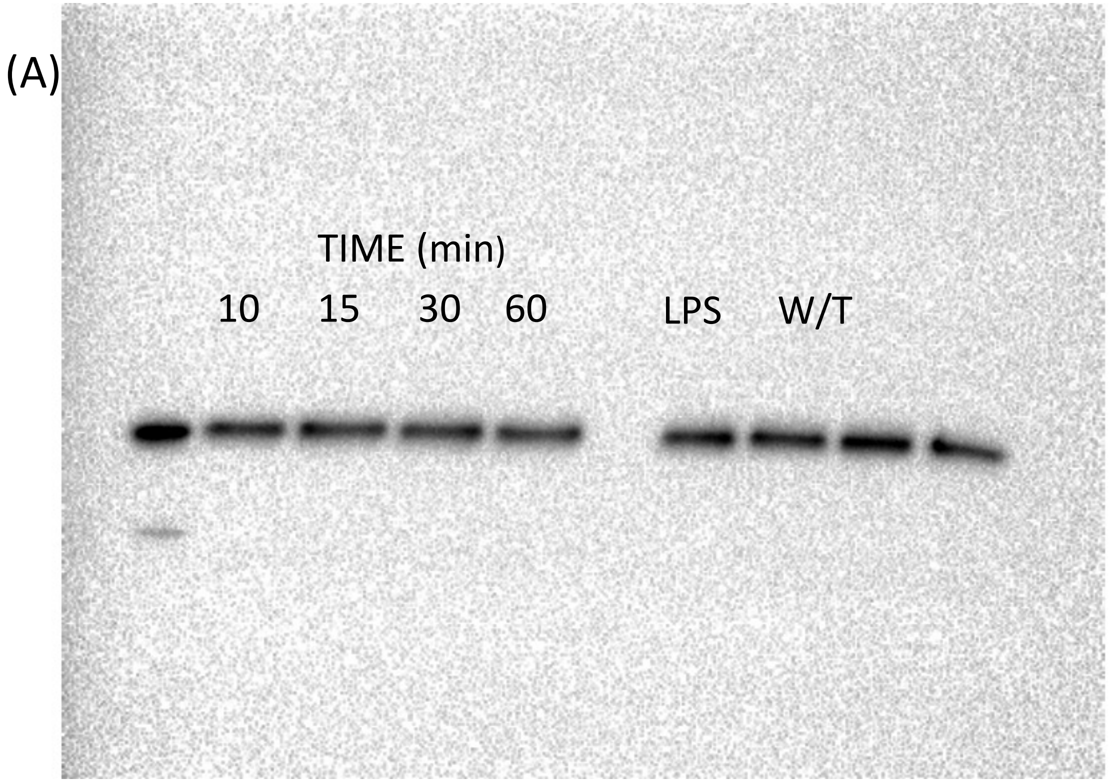

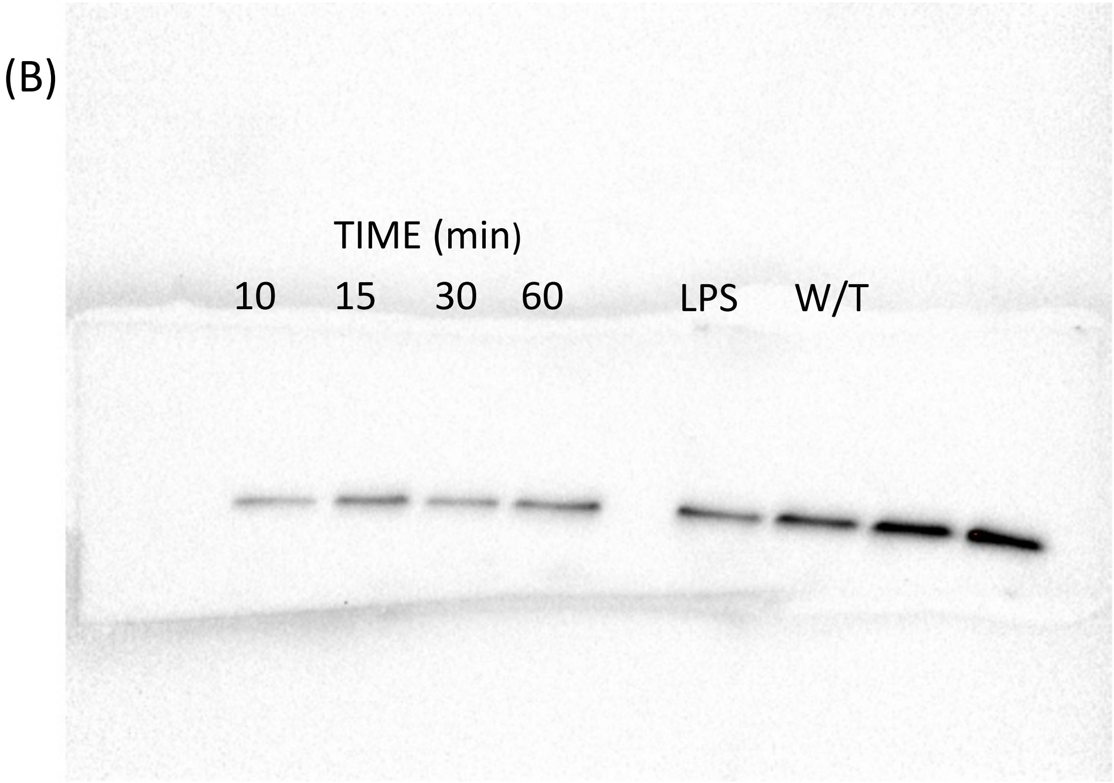

(A) Original Western blot showing kinetics of p38 MAPK of THP-1 cells treated with 40 pg/ml of VCC, incubated 10, 15, 30, and 60 min incubation without vacuolating effect. This experiment supports that VCC treatments activate transcription of p38 MAPK at 15 min. Without Treatment (W/T). (B) Original Western Blot of the β- actin protein level, considered as internal control of protein.

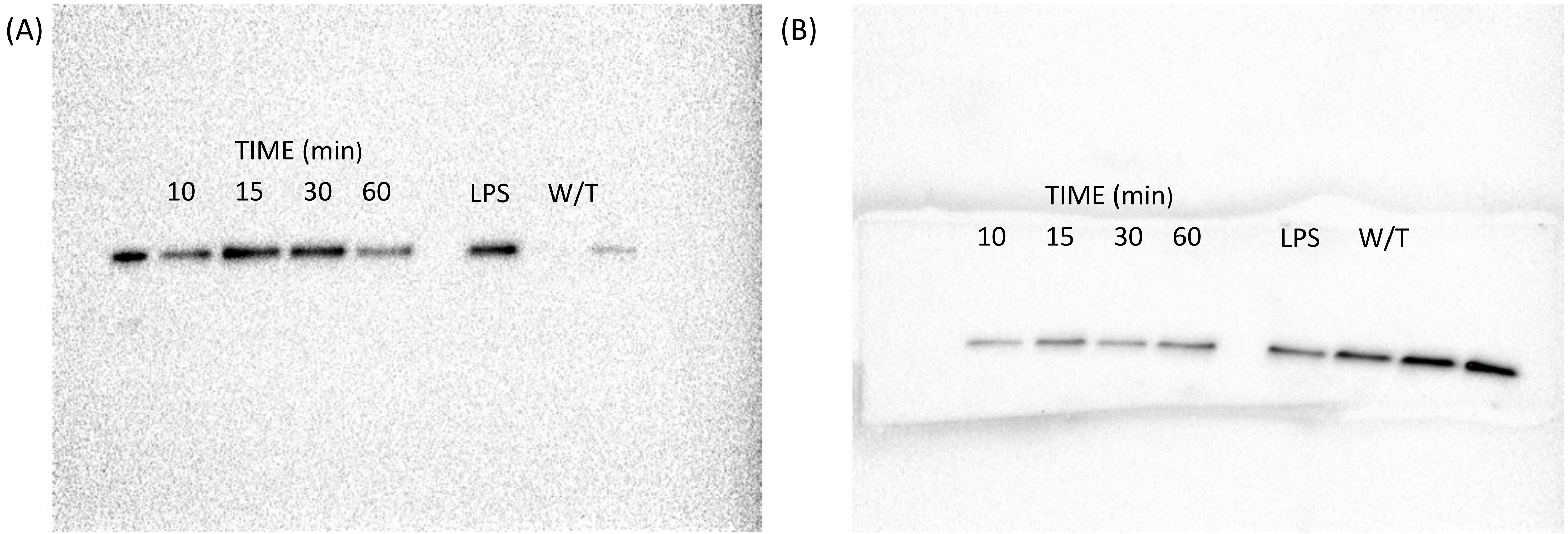

(A) Original Western blot showing kinetics of p-p38 from THP-1 cells treated with 40 pg/ml of VCC incubating 10, 15, 30, and 60 min not showing vacuolating effect. Negative control without treatment was named (W/T). This experiment supports that VCC treatments activate transcription of the phosporylated p38 MAPK at 15 min. (B) Original Western Blot of β- actin, internal control of protein.

(A)

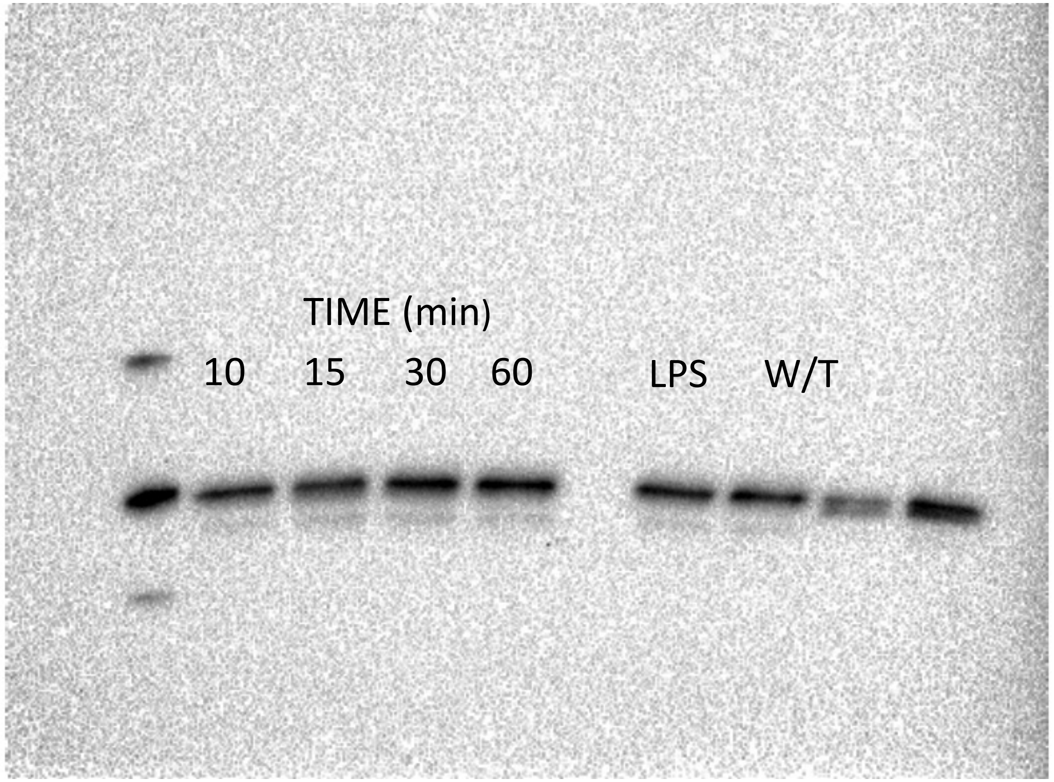

(B)

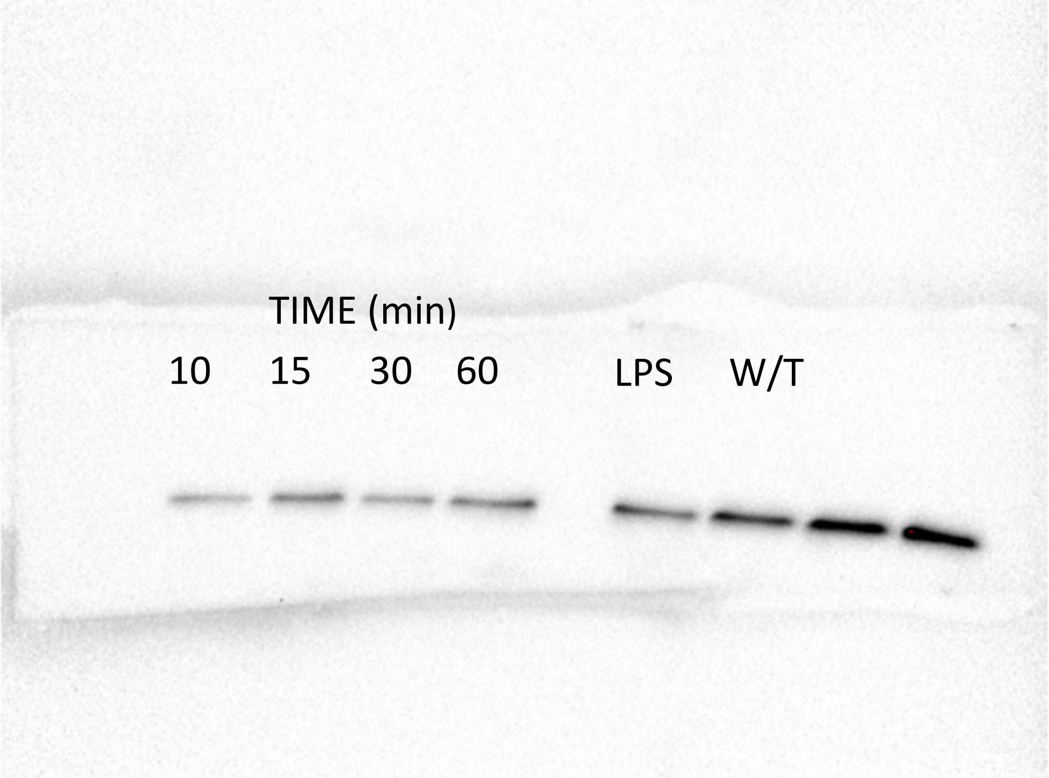

(A) Original Western blot showing kinetics of Erk MAPK activation in THP-1 cells treated with 40 pg/ml VCC, incubating 10, 15, 30, and 60 min with no vacuolating effect. Negative control without treatment was named (W/T). (B) Original Western Blot of the β- actin, an internal control of protein.

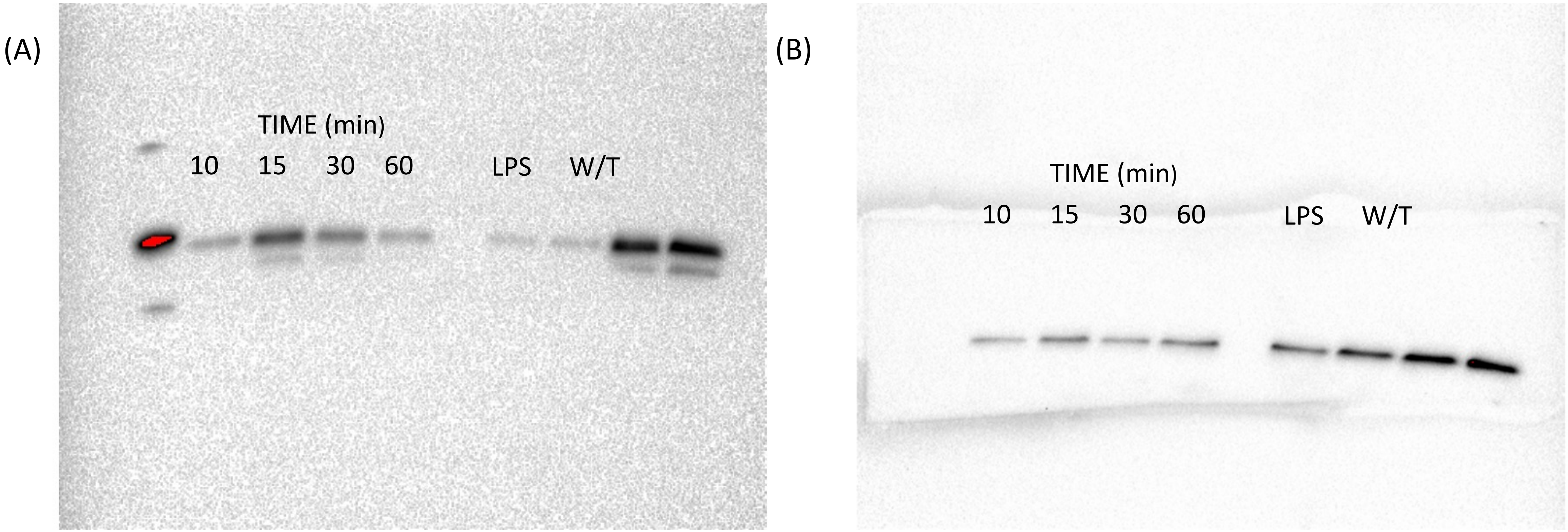

(A) Original Western blot showing p-Erk MAPK phosphorylation in THP-1 cells treated with 40 pg/ml VCC after 10, 15, 30, and 60 min incubation, with no vacuolating effect. Negative control without treatment was named (W/T).
(B) Original Western Blot of the β- actin protein, as internal control of protein.

Supplementary Information 2

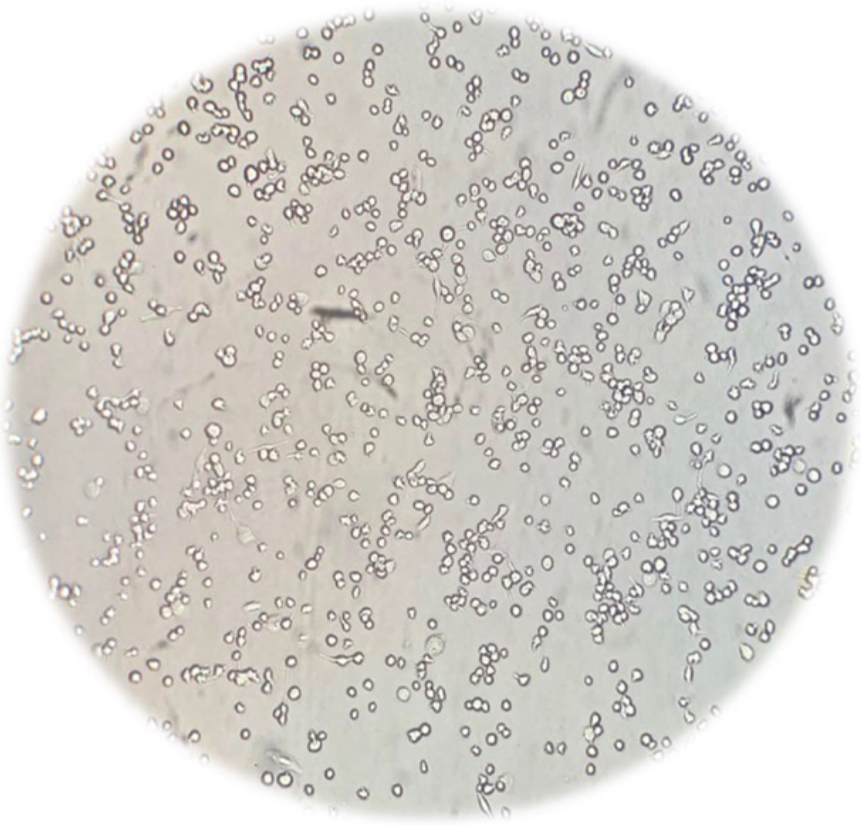

THP-1 marophages not treated with VCC (optical inverted microscope, 20x Olympus IX71). Monocytic THP-1 cells were differenciated to macrophages by incubation with phorbol 12-myristate 13-acetate (PMA) 50ng/ml for 48 hours.

20x

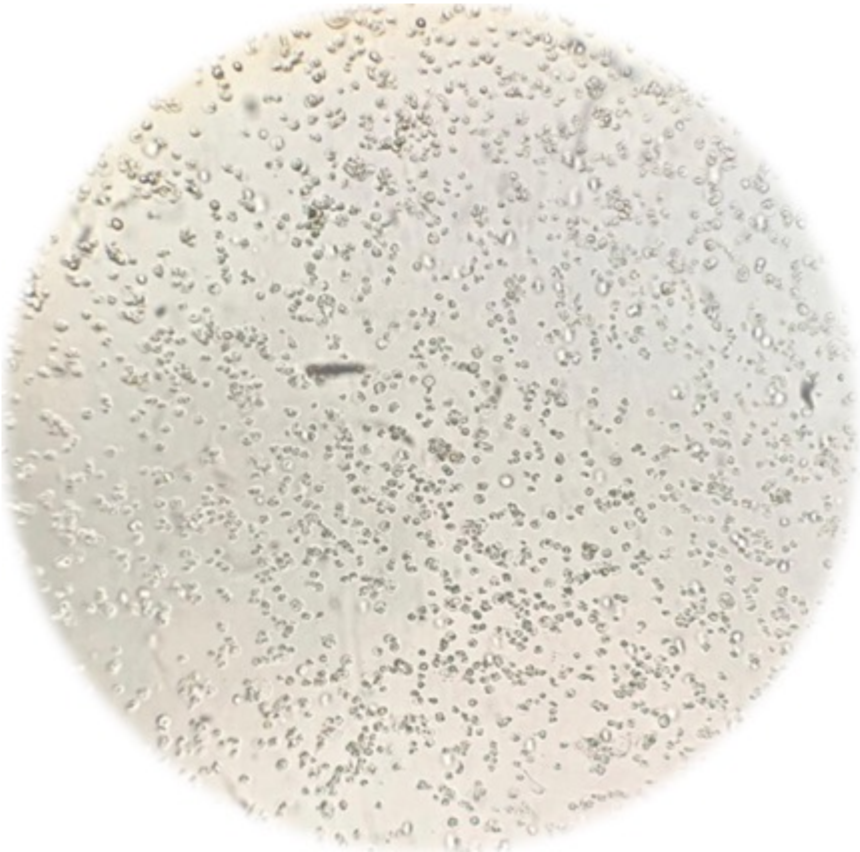

40x

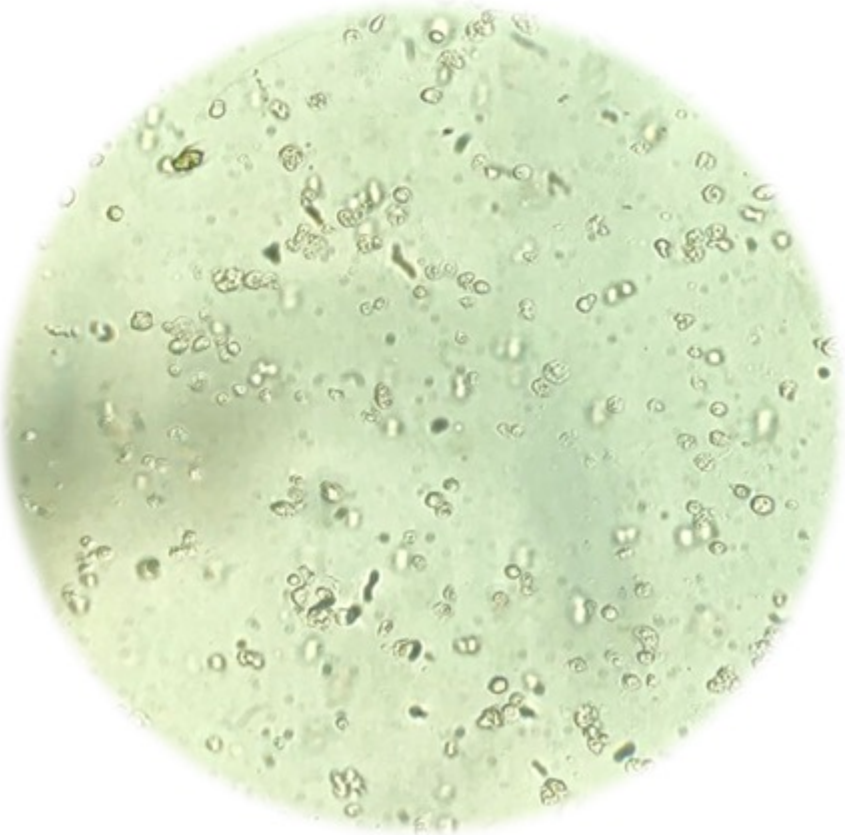

THP-1 cells previously diferenciated to macrophages then treated with 168 pg/ml *Vibrio cholerae* cytotoxin (VCC) for 6 hours. Cells were photographed under an inverted optical microscope (20X and 40X Olympus IX71). This concentration shows lytic cytotoxic effect.

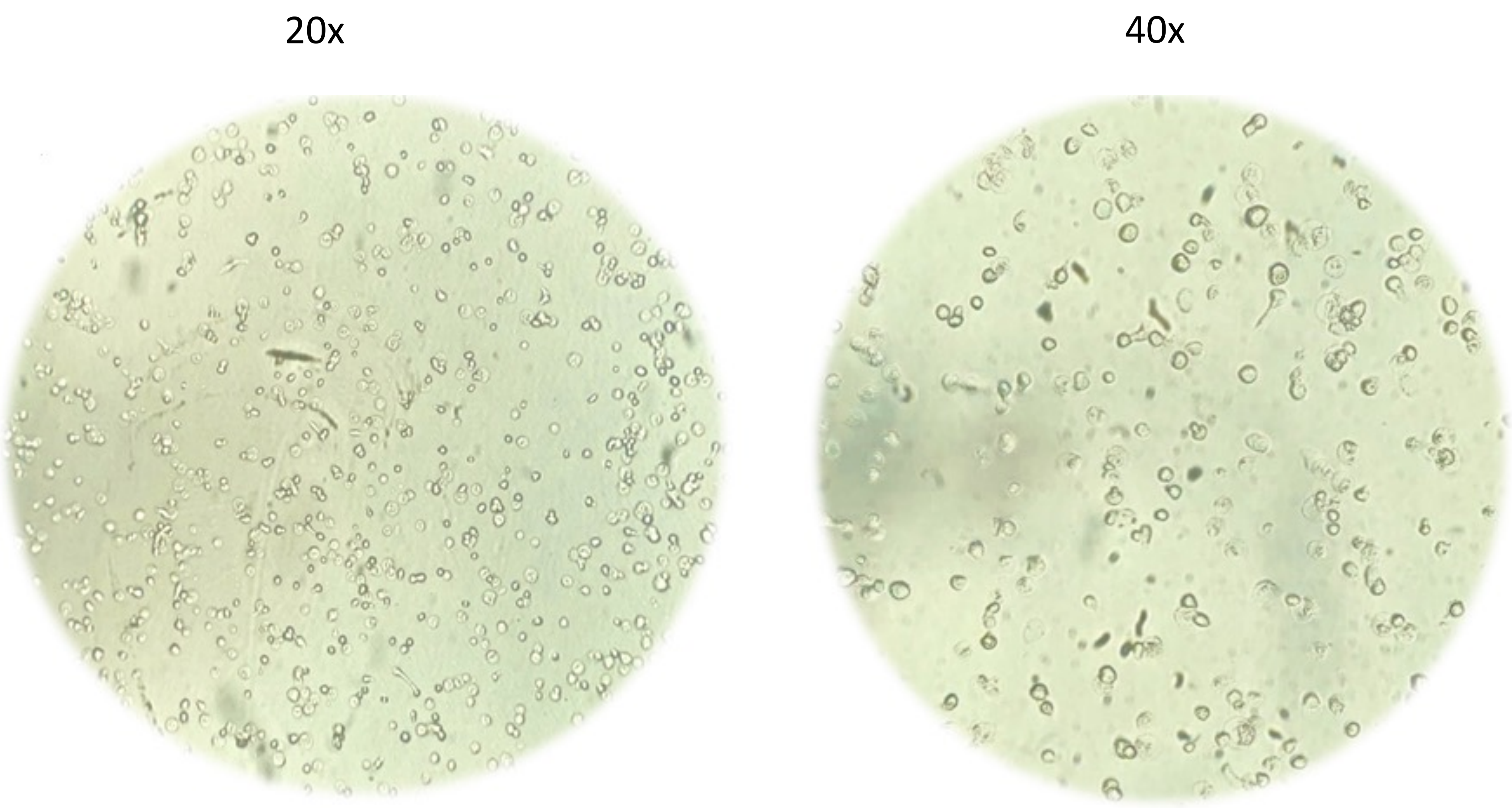

THP-1 cells previously diferenciated to macrophages then treated with 84 pg/ml *Vibrio cholerae* cytotoxin (VCC) for 6 hours. Cells were photographed under an inverted optical microscope (20X and 40X Olympus IX71). This concentration shows lytic cytotoxic effect.

20x

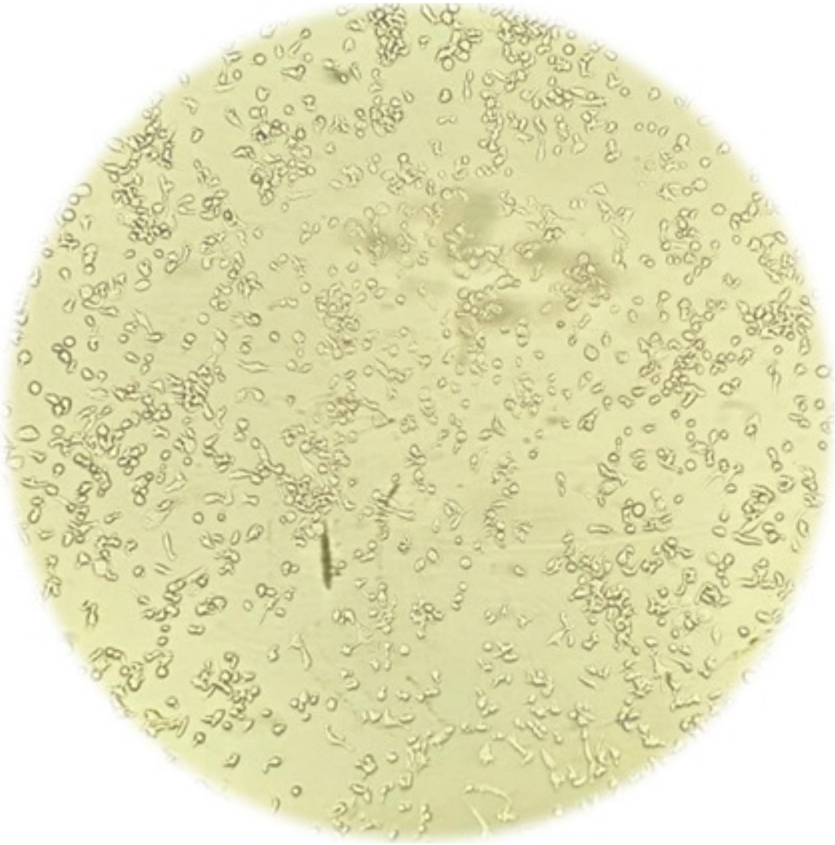

40x

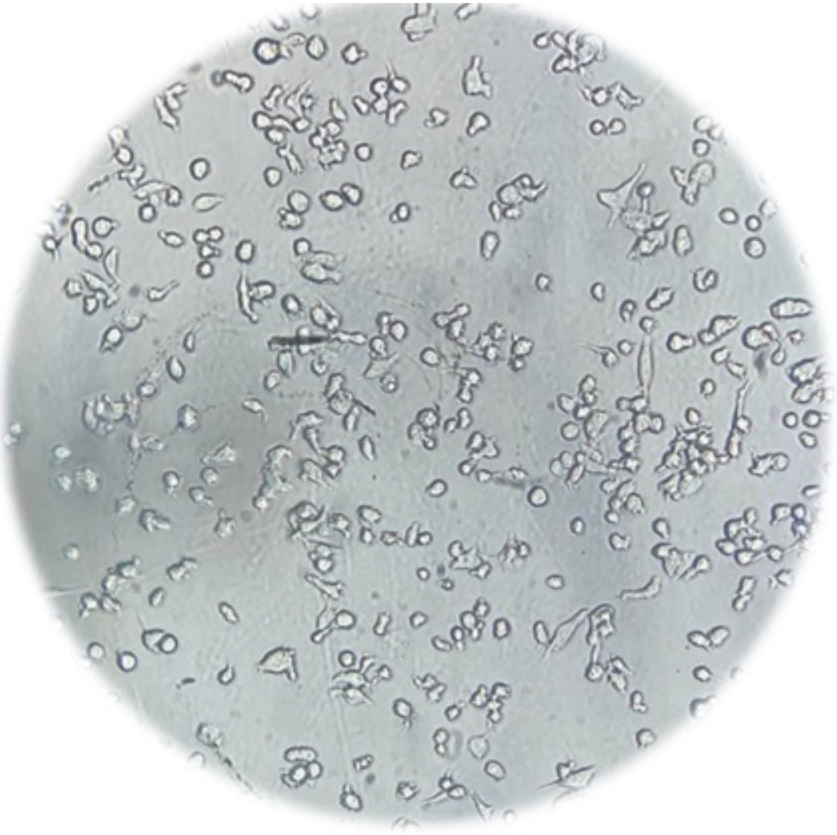

THP-1 cells previously diferenciated to macrophages then treated with 40 pg/ml *Vibrio cholerae* cytotoxin (VCC) for 6 hours. Cells were photographed under an inverted optical microscope (20X and 40X Olympus IX71). This concentration shows lytic cytotoxic effect.

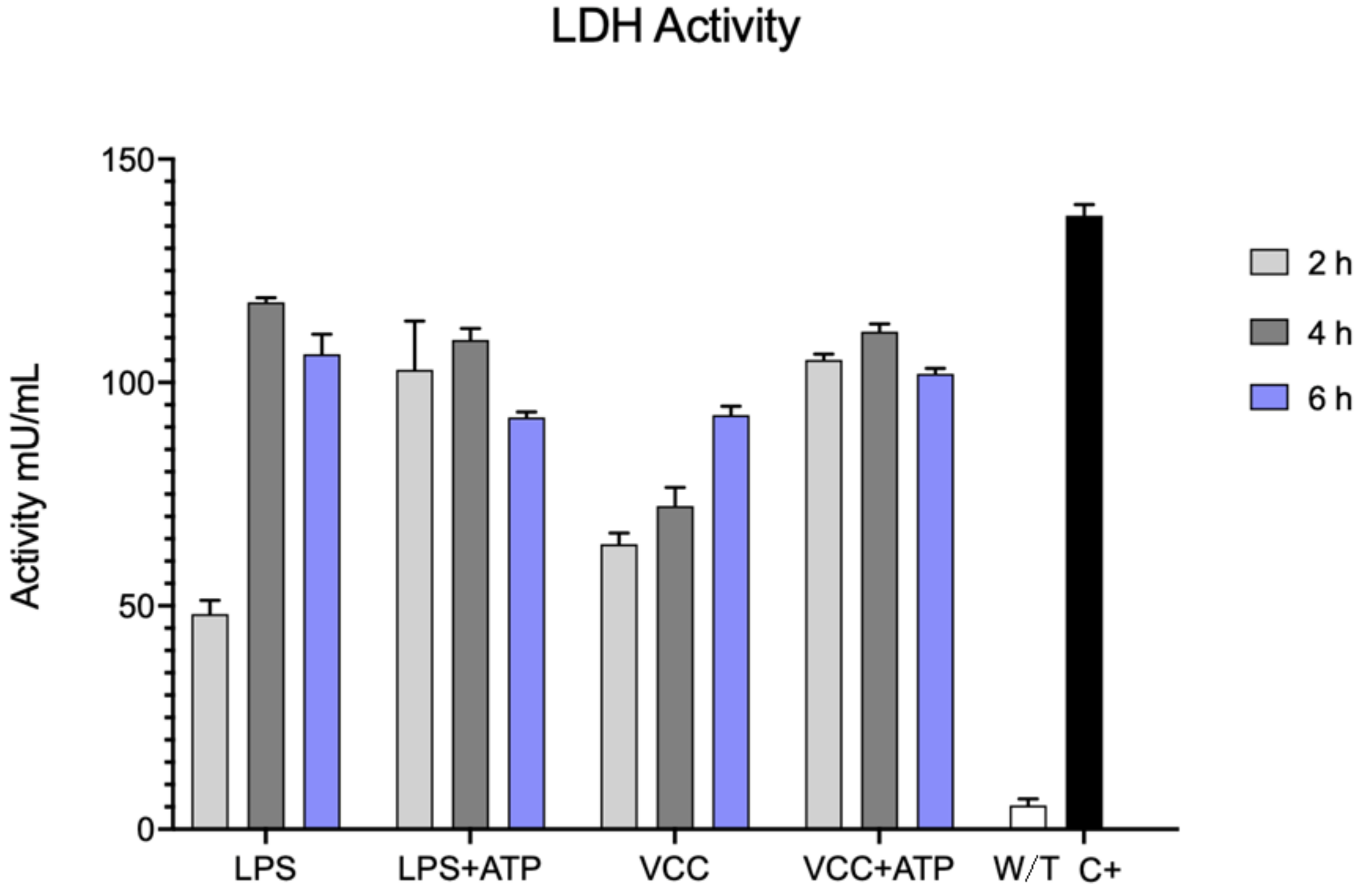

THP-1 macrophages were treated with different treatments at times of 2, 4 and 6 hours (LPS [50 ng/mL], LPS + ATP[5 mM], VCC [40pg/ml], VCC + ATP). A positive control (C+) Triton was used. Results in the graphic are representative of 3 performed experiments. W/T, without treatment. Mean ± SD. P <0.050 significantly different from the group treated with Triton (ANOVA plus the Student-Newman-Keuls method).

VCC purification steps vs rabbit polyclonal antibody anti-VCC. Western Blot

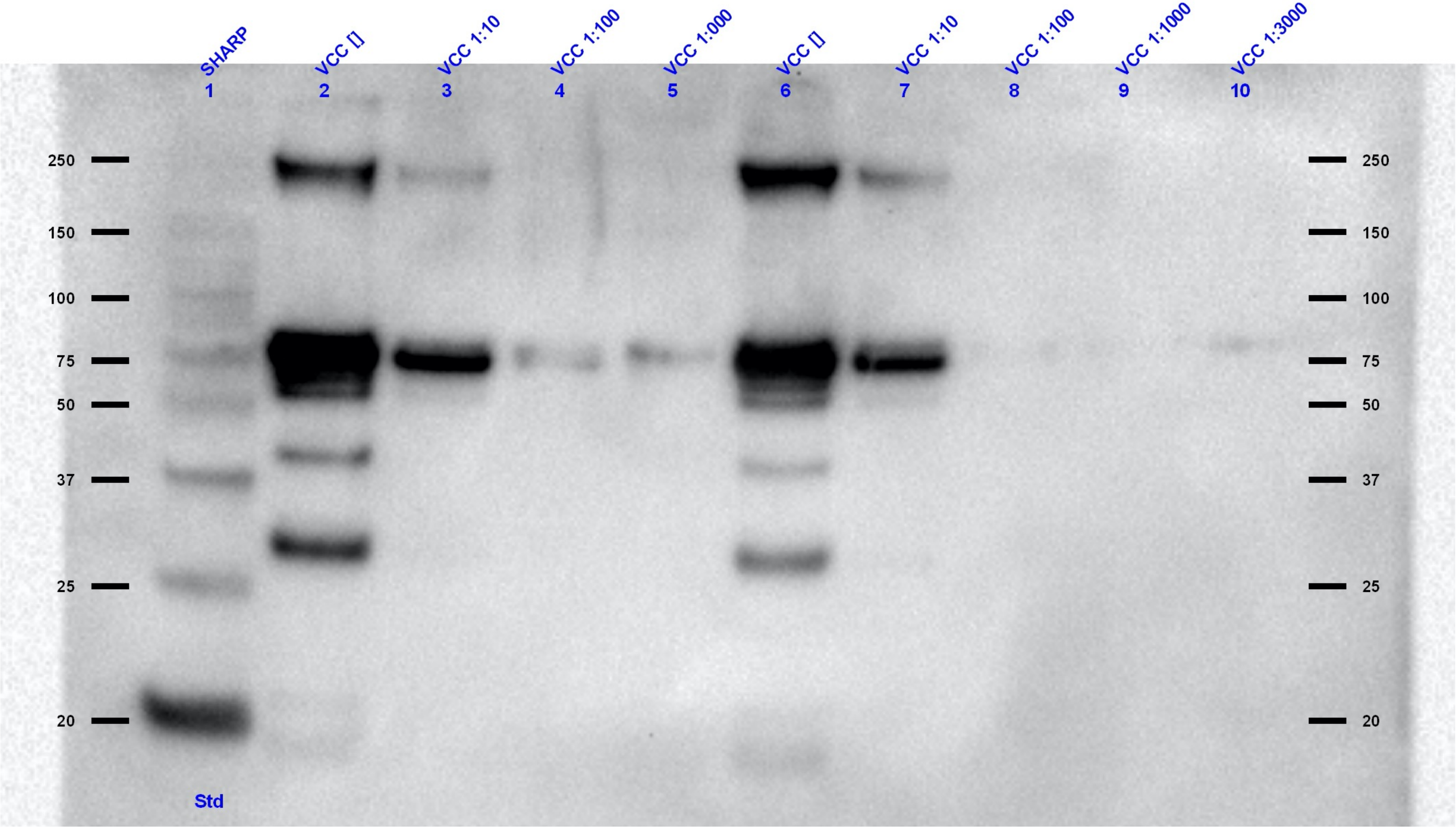

Western blot showing the purification of the VCC 65 kDa. Several steps of purification; then the purified stock serial dilutions 1:10, 1:100, 1:1000 and 1:3000. Showing that 1:3000 dilution of 40 pg/ ml still shows the VCC band of 65 kDa.
